# Research on Plant Genomics and Breeding 2.0

**DOI:** 10.3390/ijms25126659

**Published:** 2024-06-17

**Authors:** Long Jin, Zhiyong Li, Jian Zhang

**Affiliations:** State Key Laboratory of Rice Biology and Breeding, China National Rice Research Institute, Hangzhou 311400, China; jinl53719@163.com

Plant genomics and breeding is one among the several highly regarded disciplines in today’s field of biological sciences. With the development of modern biotechnology, the research approaches to examining plant gene function have become increasingly diverse. Particularly in recent years, with the accurate determination of reference genome sequences, the popularization of sequencing technologies, and the advancements made in bioinformatics, the study of gene function has become more versatile. Meanwhile, modern biotechnology plays a significant role in enhancing modern agriculture, accelerating biological breeding processes, and ensuring food security. Here, we summarize some of the genomic and breeding research works published in *IJMS* in recent years, hoping to contribute to modern breeding practices.

## 1. Genome-Wide Association Study (GWAS) Analysis

GWAS is an effective method for studying genetic variations associated with complex traits and diseases [1]. To date, numerous significant genetic loci have been identified through GWAS in both animals and plants. Nitrogen is one of the most crucial elements for plant growth. N. Ahmad et al. identified 16 candidate genes crucial for nitrogen utilization through GWAS, weighted gene co-expression network analysis (WGCNA), and RNA-Seq analysis of 327 rape (*Brassica napus* L.) germplasms, providing a genetic foundation for variety improvement and nitrogen-efficient utilization [2]. Senescence marks the final stage of plant development, accompanied by nutrient assimilation to remobilization. Liu et al. identified a homologous gene, *GhMKK9*, of the Arabidopsis cluster senescence gene *AtMKK9* through GWAS analysis of 355 upland cotton materials. They found that *GhMKK9* positively regulates plant senescence, with gene silencing delaying senescence in cotton and overexpression promoting senescence in Arabidopsis [3]. The sugar content in fresh maize stalks determines their value as green storage feed. Chen et al. conducted GWAS analysis on 188 maize germplasms, including 41 sweet maize inbred lines, 74 glutinous maize inbred lines, and 73 hybrid accessions, identifying 22 candidate genes significantly associated with stalk sugar content, 6 of which exhibited distinct expression differences between high and low sugar accessions [4]. GWAS has extensive application prospects in gene cloning, providing an important genetic basis for breeding improvement and agricultural production.

## 2. Transcriptome Sequencing Technology

RNA-seq technology enables the detection of the overall transcriptional activity of a specific species at the nucleotide level, thereby comprehensively and rapidly allowing researchers to obtain information on almost all transcripts of the species under certain conditions [5]. Szala conducted transcriptomic analysis on soybean male sterile line SXCMS5A and maintainer line SXCMS5B, revealing significant differences in expression in pathways such as pollen wall development, carbohydrate metabolism, sugar transport, reactive oxygen species (ROS) metabolism, and transcription factors, laying the foundation for understanding the mechanism of male sterility [6]. In previous studies, Zhao et al. found that *AeNAC83* in okra (*Abelmoschus esculentus*) was significantly upregulated under salt stress. Transgenic validation revealed that this gene positively regulates plant salt tolerance. Transcriptomic analysis of *AeNAC83* overexpression lines and wild-type Arabidopsis revealed that the gene enhances plant salt tolerance by affecting photosynthesis, the phenylpropane pathway, and plant hormones [7]. Wang et al. identified a leaf tip wrinkled mutant (*ltr1*) through ethyl methane sulfonate (EMS) mutagenesis in rice and found that the gene may regulate leaf development through hormone signal transduction and improve rice salt tolerance by regulating aquaporins and ion transport proteins through transcriptomic analysis. Fan et al. conducted RNA-Seq on samples treated with varying sucrose concentrations, and gene ontology (GO), Kyoto Encyclopedia of Genes and Genomes (KEGG), and Clusters of Orthologous Groups (COGs) of proteins analyses indicated that sucrose can induce cell wall loosening, extension, hydrolysis, and remodeling, induce changes in plant osmotic pressure, and affect carbohydrate transport and metabolism pathways to promote axillary bud formation [8]. RNA-Seq technology has become increasingly prevalent and essential in gene function research. It provides crucial references for a more thorough understanding of plant biology as well as potential strategies for crop improvement.

## 3. Genome Research

Complete and accurate genome assembly is crucial for a thorough understanding of the genetics and evolution of organisms. Over the past few years, a large number of genomes from different species have been published. Nurk first reported the first gapless human genome in 2022 [9], followed by an increasing number of gapless genomes of crops such as maize (MOS17) [10], rice (Nipponbare) [11], and soybean (Williams 82) [12]. The release of these genomes has facilitated further understanding of genome structure and function, providing valuable resources for genetics and genomics. Qi et al. summarized the advancements in research on the lotus (*Nelumbo nucifera*) genome and the research value of genomics in breeding. Genomic research is of great significance for studying the origin, classification, and evolution of crops, allowing explorations into the potentially valuable genes in crops and molecular breeding [13]. Xiao et al. revealed the close relationship between synonymous codon usage bias (SCUB) and genetic and epigenetic variations in rice during domestication by analyzing the codon usage bias in wild and cultivated rice [14]. Research on genomes has significantly enhanced our understanding of genome structure and function, providing valuable resources for genetics and genomics. It has made important contributions to exploring the origins, classifications, valuable genes, and molecular breeding of crops.

## 4. Molecular Marker-Assisted Breeding Research

Molecular markers are essential tools in breeding for multiple desirable traits, including Kompetitive Allele Specific PCR (KASP), Insertion and Deletion (Indel), and Simple Sequence Repeats (SSR), among others. Eltaher et al. conducted an association analysis of two KASP markers, TaDreb-B1 and 1-FEH w3, with drought tolerance in both spring and winter wheat at the seedling stage, revealing that the TaDreb-B1 marker was more effective in selecting for drought tolerance than the 1-FEH w3 allele [15]. Yang et al. employed bulked segregant analysis (BSA) pooling to map a gene controlling grain length in rice at a 96K interval and identified a KASP marker capable of effectively differentiating between grain lengths [16]. Molecular markers play a crucial role in accelerating crop breeding, and identifying molecular markers closely linked to desirable traits is of great significance for achieving genetic improvement in crops.

## 5. Gene Family Studies

A gene family is a group of genes with a common origin that encode proteins with similar structural characteristics and biochemical functions [17]. Nuclear factor YC (NF-YC) is a class of transcription factors involved in various developmental processes in higher eukaryotes such as nutrient accumulation, flowering regulation, and ABA signal response. In rice, *NF-YC8-12* are homologs with similar seed-specific expression patterns. Xu et al. demonstrated, through the creation of single mutants and quintuple mutants of *NF-YC8-12*, that these five genes redundantly regulate rice seed quality and seed germination through the abscisic acid (ABA) and gibberellic acid (GA) pathways [18]. The protein arginine methyltransferase (PRMT) family has been shown to be responsible for the methylation of specific arginine residues in proteins in plants, playing important roles in plant growth, development, and abiotic stress responses. In Arabidopsis, *AtPRMT5* plays a key role in regulating flowering time. Ling et al. identified and analyzed the PRMT family in maize, suggesting that ZmPRMT may play a potential role in responding to abiotic stress. The overexpression of the *AtPRMT5* homolog *ZmPRMT1* in Arabidopsis resulted in early flowering and increased heat tolerance [19]. Low-phosphate roots (LPRs) encode proteins containing multicopper oxidase domains. In Arabidopsis, LPR1 regulates root meristem through Fe and Pi signals, while *OsLPR5* in rice demonstrates ferroxidase activity and is necessary for normal rice growth and phosphate homeostasis maintenance. Zhao et al. analyzed the function of *OsLPR5* under salt stress, demonstrating its positive regulation of plant salt tolerance and its enhancement of ROS levels [20]. The natural resistance-associated macrophage protein (NRAMP) family is a widely distributed membrane transport protein family in plants, mainly involved in the transport of divalent metal cations such as Zn, Fe, and Cu, and plays an important role in Mn/Fe homeostasis. Zhou et al. identified the NRAMP family in betel nut, and through bioinformatic analysis of its evolutionary, cis-acting element and sequence features, they demonstrated the important role of the AcNRAMP family in betel nut in response to Zn/Fe stress based on transcriptome data under nutrient-deficient conditions [21]. The basic helix–loop–helix (bHLH) protein is one of the largest transcription factor (TF) families in eukaryotes, involved in regulating the biosynthesis of anthocyanins, flavonoids, and proanthocyanidins. Liu et al. identified 157 bHLH genes in *Ipomoea aquatica* and characterized the features and evolutionary relationships of the bHLH gene family through collinearity analysis, phylogenetic analysis, and sequence feature analysis. The subsequent transcriptome data quantification of bHLH in purple and green watercress and the analysis of 13 differentially expressed genes (DEGs) through cis-acting element analysis indicated that bHLH plays an important role in light response and plant hormone regulation, providing important insights into the role of bHLH in the anthocyanin biosynthesis pathway [22]. Determining the functions of homologous genes based on those of known functional genes can be a way to significantly enhance the study of gene functions. These studies provide important insights the research on gene families, aiding our understanding of their roles in plant growth and adaptation to environmental stress.

## 6. Conclusions and Perspective

In this review, we examined recent research articles published in *IJMS* pertaining to plant genomics and breeding. Utilizing methods such as genome-wide association analysis, transcriptome sequencing, and gene family studies, several key genes involved in nitrogen utilization, salt tolerance, and anthocyanin biosynthesis pathways were identified across different species. These findings provide important insights for understanding plant biological functions and regulatory networks, and offer valuable information for crop breeding. With the advances in modern biotechnology, numerous new technologies have emerged, including pan-genomics and synthetic biology. It is believed that, with the continuous emergence of new technologies and the maturation of existing ones, we can enrich our understanding of the genetic basis of crops, and breeding methods will become more diverse and streamlined.
